# Can this data be saved? Techniques for high motion in resting state scans of first grade children

**DOI:** 10.1016/j.dcn.2022.101178

**Published:** 2022-11-17

**Authors:** Jolinda Smith, Eric Wilkey, Ben Clarke, Lina Shanley, Virany Men, Damien Fair, Fred W. Sabb

**Affiliations:** a Robert and Beverly Lewis Center for Neuroimaging, University of Oregon, Eugene, OR, USA; b Brain & Mind Institute, Western University, London, Ontario, Canada; c Center on Teaching and Learning, University of Oregon, Eugene, OR, USA; d School Psychology, College of Education, University of Oregon, Eugene, OR, USA; e Psychiatry and Behavioral Neuroscience, Oregon Heath & Science University, Portland, OR, USA; f Prevention Science Institute, University of Oregon, Eugene, OR, USA

**Keywords:** Fmri, Motion, Artifact, Resting-state, Independent component analysis

## Abstract

Motion remains a significant technical hurdle in fMRI studies of young children. Our aim was to develop a straightforward and effective method for obtaining and preprocessing resting state data from a high-motion pediatric cohort. This approach combines real-time monitoring of head motion with a preprocessing pipeline that uses volume censoring and concatenation alongside independent component analysis based denoising. We evaluated this method using a sample of 108 first grade children (age 6–8) enrolled in a longitudinal study of math development. Data quality was assessed by analyzing the correlation between participant head motion and two key metrics for resting state data, temporal signal-to-noise and functional connectivity. These correlations should be minimal in the absence of noise-related artifacts. We compared these data quality indicators using several censoring thresholds to determine the necessary degree of censoring. Volume censoring was highly effective at removing motion-corrupted volumes and ICA denoising removed much of the remaining motion artifact. With the censoring threshold set to exclude volumes that exceeded a framewise displacement of 0.3 mm, preprocessed data met rigorous standards for data quality while retaining a large majority of subjects (83 % of participants). Overall, results show it is possible to obtain usable resting-state data despite extreme motion in a group of young, untrained subjects.

## 1 Introduction

Functional MRI of the human brain at rest represents a growing and significant share of published neuroimaging studies over the past twenty years (Lowe, 2010, Lowe, 2012, Specht, 2020, van den Heuvel and Hulshoff Pol, 2010). While task-based imaging examines neural activity evoked as a response to experimental tasks, resting state fMRI (rs-fMRI) examines temporal correlations in spontaneous (or “intrinsic”) BOLD signal across the brain, typically while the participant is resting. This technique has been used extensively across a wide age range, from fetal MRI in utero and preverbal infants (Graham et al., 2015, Schöpf et al., 2012, Seshamani et al., 2016) to adults and the elderly (Ferreira and Busatto, 2013), and to understand both typical and atypical development (Finn et al., 2017, O’Connor and Zeffiro, 2019, Rashid and Calhoun, 2020, Shen et al., 2017, Specht, 2020). Resting state fMRI has several advantages over task-based fMRI that make it such a widely applicable tool. Since there is no behavioral component, an rs-fMRI protocol is relatively easy to administer, requires no additional equipment or participant training, and avoids confounds typically introduced by individual or group differences in task behaviors.

However, this expanded use of fMRI has also introduced challenges. Artifacts in the acquisition of rs-fMRI data due to motion remain a significant impediment to studying critical processes in both younger children (Satterthwaite et al., 2012) as well as those who may have atypical trajectories or symptomatology that makes them more likely to move during data acquisition (e.g. attention disorders (Epstein et al., 2007; Fair et al., 2013) or autism spectrum disorder (Cox et al., 2017)). Furthermore, the analysis techniques used to analyze rs-fMRI data are often more susceptible to the impacts of motion artifacts than task-based fMRI (Power et al., 2015). While much progress has been made in correcting artifacts caused by head motion in functional MRI data (Burgess et al., 2016, Caballero-Gaudes and Reynolds, 2017, Ciric et al., 2017, Dipasquale et al., 2017, Parkes et al., 2018, Power et al., 2015), applying these methods to cases of extreme head motion remains challenging. Real-time monitoring of head motion can allow the researcher to collect sufficient low-motion frames over multiple runs, but one is still left with the question of how to select the “best” data for each subject, and how to best combine data from multiple runs into one “clean” dataset.

Volume censoring is the identification and removal of outlier volumes using relative motion metrics such as framewise displacement or relative root mean squared (RMS) intensity differences (Power et al., 2012, Power et al., 2014). After censoring and appropriate preprocessing, data from multiple runs may be concatenated for further processing (Power et al., 2014, Yan et al., 2013). Because censoring and concatenation both change the temporal nature of the data, care must be taken when combining censoring and concatenation with other processing steps. For example, low-pass filtering will no longer be valid after censoring, but low pass filtering before censoring will contaminate the data with artifacts from the censored volumes. Likewise, regression of nuisance variables such as motion parameters and physiological signals must be performed carefully. Either the regression model should have any rows corresponding to censored volumes removed (Caballero-Gaudes and Reynolds, 2017), or the censored volumes should be replaced by interpolated data that will be removed later (Power et al., 2014). In this work, we choose to forgo temporal filtering and nuisance regression, instead relying on independent component analysis (ICA) based denoising to remove any artifact remaining after censoring.

Independent component analysis automatically separates temporal data into a number of maximally independent spatial components (Beckmann and Smith, 2004, McKeown et al., 1998). Artifactual components may be regressed out, leaving a cleaned signal behind (Thomas et al., 2002). Components may be labeled as noise or signal either by hand (Griffanti et al., 2017), automatically based on spatial and/or temporal features (Pruim et al., 2015), or using a trained classifier (Salimi-Khorshidi et al., 2014). Automatic identification is appealingly easy to implement but may not perform well on data that is characteristically different from the data that the methods were originally developed for. Training a classifier can be a subjective and time-consuming process, but may perform better if the training data is well-chosen. A well-trained dataset will include noise sources that are commonly regressed out, such as head motion and physiology, reducing the need for additional regression of these sources. In fact, regression of these noise sources in addition to ICA-based denoising may not only be redundant, but undesirable, as the use of too many regressors can remove signal as well as noise (Bright et al., 2017, Bright and Murphy, 2015).

In this manuscript, we provide one potential process for acquiring and cleaning high-motion resting state data typically observed in pediatric cohorts. Our aims were to use real-time fMRI monitoring software to maximize the number of subjects with sufficient data for further analysis, to evaluate a simple preprocessing pipeline that includes censoring and ICA-based denoising, and to determine whether pipeline parameters exist that offer satisfactory performance in high-motion pediatric subjects. We evaluated the success of our preprocessing performance by analyzing the correlation of head motion with two hallmark indicators of data quality in the resting-state literature: (1) temporal signal-to-noise ratio (tSNR) and (2) functional connectivity. We then compared these data quality indicators across several exclusion thresholds related to a subject’s average movement to determine the pipeline’s impact on samples with varying degrees of movement. While preliminary, we believe this approach allows for studying children and other high-motion groups that would otherwise be excluded as research participants. Including high-motion subjects in fMRI studies prevents gaps in our knowledge of early development and of less typically developing children.

## 2 Methods

### 2.1 Participants

The sample for the current study is comprised of students in the first three cohorts of a multi-year National Science Foundation (NSF; DRL 1748954 & DRL 1660840) funded study aimed at examining cognitive and neural correlates of first grade mathematics development. Year 1 and year 2 participants were recruited from schools that participated in a large-scale efficacy trial of a first-grade mathematics intervention funded by the Institute of Education Sciences (IES; R324A160046). Year 3 participants were recruited from schools who continued to implement first grade mathematics intervention after the conclusion of the IES study. In all 121 students participated in years 1–3 of this study. Parental consent was obtained for all subjects in accordance with a protocol approved by the University of Oregon institutional review board. In the full sample, 55 % reported their biological sex as male. Additionally, 1 % of participants identified as Asian, 1 % identified as Black, 4 % identified as Native Hawaiian/Pacific Islander, 94 % identified as White, 11 % identified as Hispanic or Latino, and 6 % were reported as more than one race. Of these students, 12 % were eligible for special education and 5 % met criteria for limited proficiency in English. One hundred and eight children completed at least one MRI session. Of the 108 children that completed an MRI session, 101 (54 M, 47 F) also had usable T1 anatomic scans and were included in further analysis. If a child completed additional sessions, the best T1 from any session was used. Children ranged in age from 6 to 8 years, with a mean age of 6.8 years.

### 2.2 Procedure and data acquisition

Children underwent mock scanning immediately prior to each session and were instructed on the importance of holding still, but otherwise had no special training. Imaging data was collected on a Siemens 3T Skyra, consisting of a localizer, a T1-weighted anatomic (GRAPPA accelerated MPRAGE, TR = 2500 ms, TE = 3.43 ms, TI = 1100 ms, flip angle = 7°, 1 mm isotropic resolution), and one or more BOLD functional scans. Functional imaging was performed with the C2P multiband EPI sequence distributed by the Center for Magnetic Resonance Research (CMRR) at the University of Minnesota (https://www.cmrr.umn.edu/multiband/), with TR = 780 ms, TE = 32 ms, flip angle = 55°, 2.5 mm in-plane resolution, 2.5 mm slice thickness, and multiband acceleration factor = 3. Two versions of the T1-weighted scan were used: one with an acceleration factor of two and total duration of 5:56 (62 subjects), and one with an acceleration factor of three and a total duration of 3:28 (39 subjects).

**Fig. 1:**
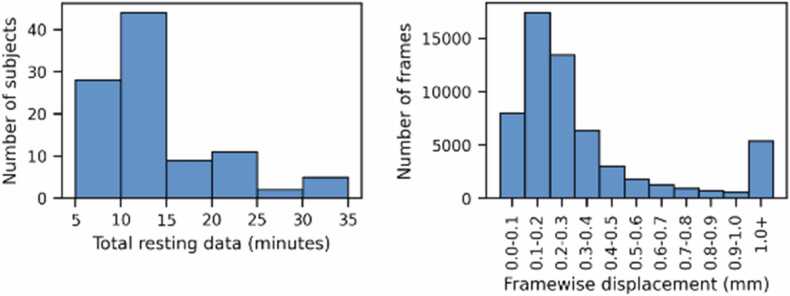
Histogram of the total amount resting state data obtained from all subjects, and of the framewise displacement for all frames of resting state data collected.

Subject motion was monitored during fMRI using Framewise Integrated Real-time MRI Monitoring (FIRMM, https://firmm.io/). Additional scans were performed until FIRMM reported that we had obtained at least 4 min of total (non-contiguous) data comprised of frames with less than 0.4 mm of framewise displacement, until the subject requested an end to the session, or until the researcher decided that the subject was unlikely to reach the targeted FIRMM threshold. Framewise displacement (FD) in this instance is defined as a weighted average of rotational and translational displacements (Power et al., 2012). FIRMM does not report on the length of retained segments between frames outside of the targeted threshold, and in later processing no minimum length for retained segments was imposed. Each of the 101 subjects with a usable anatomic scan from any session also completed at least 4 min total of resting state. Eight of those subjects did not meet the desired FIRMM targets but were included in further analysis in order to investigate more lenient censoring thresholds. One subject was later excluded because of susceptibility artifacts in the data. For the 100 subjects included in further analysis, resting state data was collected over the course of 1–7 scans. If at least 4 min of resting state data within the framewise displacement threshold were collected, subjects were given the option of performing one or more task-based functional scans; 58 subjects chose this option. The total time from the beginning of the first scout to the end of the last resting state scan ranged from 14 to 164 min, with a mean of 53 min. This included breaks from scanning and time out of the magnet; for example, the subject with the longest session had 33 min of actual scan time and 130 min of break time between scans. Subjects were allowed to end the session at any time, and sessions were ended immediately if the subject appeared to be unhappy or distressed. Actual resting data collected ranged from 5 to 37 min, with a mean of 12 min (Fig. 1). The mean FD for all resting scans combined was 0.50 mm, median 0.22 mm. The maximum FD measured in any scan was 35 mm.

### 2.3 Functional data pipeline

DICOM data was converted to nifti format using MRIConvert (Li et al., 2016). Three initial volumes were discarded from each set after we determined that the magnetization had not yet reached a steady state for these short TR acquisitions. FSL Motion Outliers was used to determine the framewise displacement at every point for each run (Jenkinson et al., 2012, Smith et al., 2004). Motion correction was calculated and applied prior to FD calculation, but the data fed into the next step was not motion corrected. Four different censoring thresholds were compared, including 0.25, 0.3, 0.4, and 0.5 mm. Volumes whose FD exceeded the threshold were removed from the data, but adjacent volumes were not. The censored runs for each subject were then concatenated into single long runs.

**Fig. 2:**
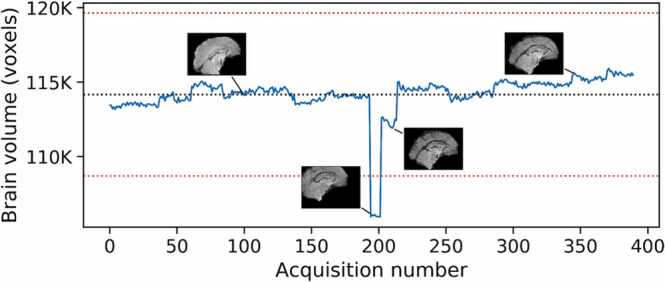
Deviations from mean brain volume used to flag problematic functional frames. The black dotted line shows the mean brain volume for all frames; the red dotted lines show ± 4 std dev from the mean. Insets show the mid-sagittal view of the extracted brain at four time points. Frames whose brain volumes deviated from the mean by more than four standard deviations were rejected.

Because of the high degree of motion in these subjects, occasionally there would be volumes that, while free of artifact, were nevertheless unusable because the subject had moved partially out of the imaging field of view. In order to flag and remove these volumes, we removed any for which the total number of brain voxels was substantially lower than the mean all functional scans. This step was done by performing brain extraction on each volume of the concatenated data, calculating the total number of brain voxels in each volume, and rejecting any volumes more than four standard deviations away from the mean number of brain voxels across all volumes (Fig. 2). The python scripts used for volume censoring and for identification of outlier volumes is available at https://github.com/Jolinda/lcni_motion_scripts.

If a subject’s T1 weighted image from the first session was judged by visual inspection to be of too poor quality to be used, the T1 image from the second session was used. This was true for seven subjects, for whom the second session occurred on average 137 days after the first. Any subject without a T1 judged to be sufficiently high-quality was excluded from further analysis (an additional seven subjects). T1 scans were skull-stripped using FSL’s brain extraction tool (BET). BET’s performance on pediatric brains was improved by adding a fractional intensity threshold gradient in the anterior to posterior direction; with this modification we were able to perform fully automated brain extraction in all anatomic scans. The skull-stripped anatomic scans were registered to a 2 mm MNI template using nonlinear transforms and FSL’s FNIRT.

The censored and concatenated data were motion corrected, slice-time corrected, brain extracted, and registered to each subject’s chosen T1 scan. Each functional volume was intensity normalized to 10,000 units across brain voxels to account for differences in the arbitrary intensity scale used in different functional runs.

FSL’s Melodic was used on the corrected concatenated data to generate 60 ICA components. FMRIB's ICA-based Xnoiseifier (FIX) (Griffanti et al., 2014, Salimi-Khorshidi et al., 2014) was used together with a custom dataset, described below, to identify and remove noise components using the “aggressive” option. Finally, each concatenated dataset was trimmed to the first four minutes. Four minutes has been shown to be a sufficient amount of data for observing functional networks (Van Dijk et al., 2010), but whether it is adequate for a given study will depend on the effect size under consideration and the quality of the data. For a scan duration of 4 min with TR = 0.78 ms, the required temporal signal to noise (tSNR) should be approximately 50 in order to detect an effect size of 1 % with p = 0.05 (Murphy et al., 2007). Although longer scan durations have been shown to improve reliability (Birn et al., 2013), practical considerations with this population make it difficult to collect as much data as one might wish. As seen in Fig. 1, more than 70 % of subjects required more than eight minutes of data before four minutes of “good” frames were collected, and almost 20 % required more than 16 min. In this study we used the lowest possible scan duration in the hopes of obtaining data from as many participants as possible. Trimming each dataset to the same length was performed to avoid biasing the results towards the low-motion subjects; however, other work has shown that the impact of trimming depends on the analysis techniques used and such trimming may not be necessary in all cases (Power et al., 2014).

### 2.4 Custom ICA denoising training set

Data from twenty subjects was used to create a custom training set for ICA denoising with FIX. Subjects were chosen for inclusion in the training set if they lacked a useable T1 scan or if they only completed one of two longitudinal sessions. This work includes data from subjects who only completed the first session even if they were also included in the training set data (six subjects). As the current work does not evaluate the performance of automatic classification vs hand-labeling, the effect of using slightly overlapping datasets for training and test was felt to be a minor concern. The training data underwent an earlier version of our censoring approach, in which the data was broken into blocks of at least 45 s duration with a framewise displacement of no more than 1.4 mm. After censoring and concatenation the data runs averaged 5.9 min, with a maximum length of 7.8 min and a minimum length of 4.2 min. Data sets were not trimmed to the same length. Due to a coding error the initial frame in each block had a FD greater than or equal to 1.4 mm, but retaining these high FD peaks guaranteed that the training set would include any artifacts related to the concatenation process, as well as artifacts related to high motion. ICA components from the training set were hand-labeled by JS as either signal or noise, using the guidelines in Griffanti et al. (2017). These guidelines outline spatial, temporal, and spectral features that are characteristic of either signal or noise. Examples of features indicative of signal components include high overlap with brain grey matter regions, spatial patterns with no relationship to acquisition parameters, and predominantly low frequency content without sudden large jumps in the temporal domain.

**Fig. 3:**
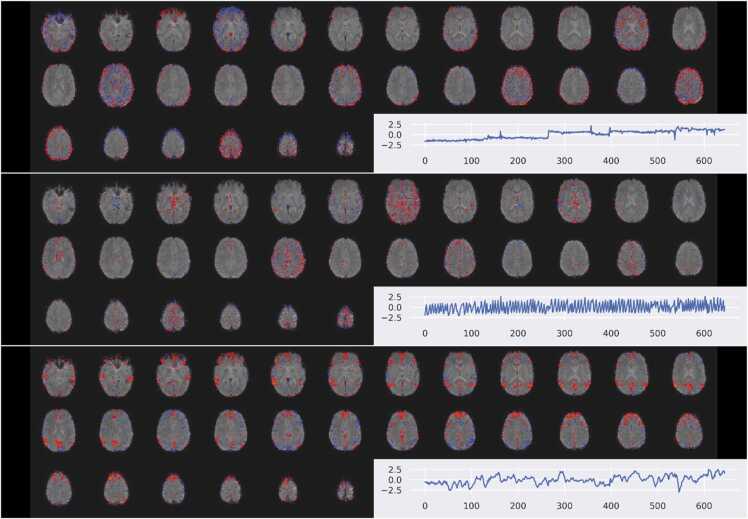
Three ICA components from a single subject with associated time courses. The top two components may be identified as noise components by both spatial and temporal characteristics. Spatially, they both show clusters predominately in non-grey matter locations, with a periodicity related to the acquisition parameters. The time course of the first component shows several sudden jumps, and the second appears to be predominately high frequency. In contrast, the final signal component has a regular, low frequency time course, is predominately in grey matter areas, and does not show any spatial relationship to acquisition parameters.

Fig. 3 illustrates three example spatial components with their associated time courses from a single subject. The top component is likely the result of sudden motion: the time course has several sudden jumps, and the spatial component is predominantly in a ring around the outer surface of the brain. The middle component features high-frequency content in non-grey matter areas. Both the top and middle components are also clearly dependent on acquisition parameters: the spatial component is strongest every fourth slice. This periodicity is due to our use of an interleaved acquisition with multiband factor = 2, and illustrates the utility of using training data with appropriate acquisition parameters, rather than more general datasets. The lowest component is identified as signal: it’s predominately in grey matter areas, has a fairly regular time course, and does not have a spatial pattern related to acquisition parameters.

### 2.5 QC-FD correlations

In order to judge the effectiveness of our preprocessing pipeline we asked whether various quality control metrics correlated with head motion, and whether the pipeline reduced this correlation. We looked at two different QC metrics: (1) functional connectivity and (2) temporal signal-to-noise ratio (tSNR), correlated with the mean FD of the censored data before further preprocessing.

Functional connectivity was measured using a subset of 67 out of 333 cortical regions defined by Gordon et al. (2016), chosen because they contained at least 5 voxels of grey matter across all 101 subjects. Mean time series signals were averaged over all voxels in each region, and the Pearson correlation coefficient was calculated for every pair of nodes, resulting in a set of correlation values for each intra-node pair, or edge. We then compared the proportion of edges significantly correlated with FD (p < 0.05) across censoring thresholds to assess pipeline performance. With no impact of movement on the data, the percentage of edges significantly correlated with FD would be at chance. Further, movement artifacts have been shown to inflate short-range functional connectivity compared to longer distances (Van Dijk et al., 2012; Power et al., 2012, 2015). To address this concern, we looked at the strength of correlation as a function of intranodal distance. Successful noise correction should result in no distance-dependence of functional connectivity correlations. It should be noted that the use of this metric may be problematic because of the possibility of actual differences in functional connectivity between high- and low-motion subjects (Williams et al., 2022). Because of this we added a second metric for comparison, tSNR.

TSNR is a widely used metric of overall fMRI image quality and time course stability (Murphy et al., 2007) but has particular relevance for rs-fMRI since results are driven by temporal correlations, and therefore are more susceptible to the impact of noise over time. TSNR was defined as the mean whole-brain signal divided by the standard deviation across time (Parrish et al., 2000). We compared tSNR across various pipeline settings to measure the efficacy of our preprocessing pipeline in removing motion-related noise at different censoring thresholds with and without the FIX procedure applied.

Fully preprocessed, denoised, and trimmed data were transformed into standard MNI space. The standard space images were concatenated and fed into a group-level, 40-component analysis using FSL’s MELODIC. A separate group analysis was performed for each of the four censoring thresholds tested.

## 3 Results

### 3.1 Subject exclusion by framewise displacement threshold

**Fig. 4:**
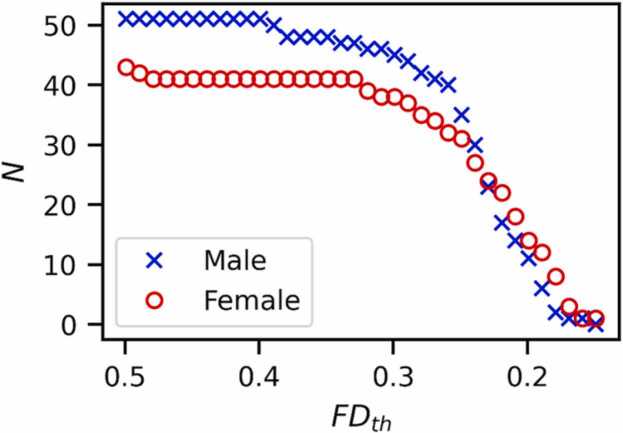
Number of subjects with at least four minutes of data after censoring at various framewise displacement thresholds (FD_th_). The number of remaining subjects falls sharply at thresholds less than 0.3 mm, and male subjects are lost more rapidly than female subjects. The initial subject pool was majority male, becoming majority female for FD_th_ < 0.24 mm. Mean framewise displacement was significantly higher for male vs female subjects: 0.40 ± 0.09 mm for females, 0.48 ± 0.10 mm for males.

In order to assess the effect of censoring on subject exclusion, we looked at the number of subjects that could be included in further analyses at various FD censoring thresholds (FD_th_). Fig. 4 shows the number of male and female subjects that would have four minutes of data in our sample as a function of FD_th_, our inclusion criteria for further analysis. As expected, for thresholds at or above the same thresholds set in FIRMM (0.4 mm), nearly all subjects had enough data for further analysis (51 out of 54 males, and 42 out of 47 females), reflecting the eight subjects whose sessions were ended early. As stricter thresholds were applied, not only did the total number of included subjects fall dramatically, but the male/female ratio shifted towards more female subjects. This is unsurprising given our cohort, as there was a significant difference in mean FD between males and females, with mean FD = 0.40 ± 0.09 mm for females, 0.49 ± 0.10 mm for males. In order to maintain our original group demographics we limited our FD censoring thresholds to values in which at least 66 % of all subjects remained in the sample. We focused on four values of FD_th_ for further analysis: 0.25 mm (66 % of subjects), 0.3 mm (83 % of subjects), 0.4 mm (92 % of subjects), and 0.5 mm (94 % of subjects). These four thresholds were then compared by looking at the correlation of our two quality control metrics, tSNR and functional connectivity, with mean framewise displacement at each threshold.

We were similarly concerned that excluding subjects due to motion would bias our sample towards older participants, as head motion is known to be correlated with age (Satterthwaite et al., 2013). However, we saw no such effect, and also saw no significant correlation between mean framewise displacement and age in our sample. This is consistent with the results of Achterberg and van der Meulen, who saw only a small association between age and percentage of scans meeting stringent quality thresholds in a similarly aged cohort (Achterberg and van der Meulen, 2019). This lack of correlation is likely due to the relatively narrow range of ages in our population.

### 3.2 Temporal signal to noise ratio correlation with FD

**Fig. 5:**
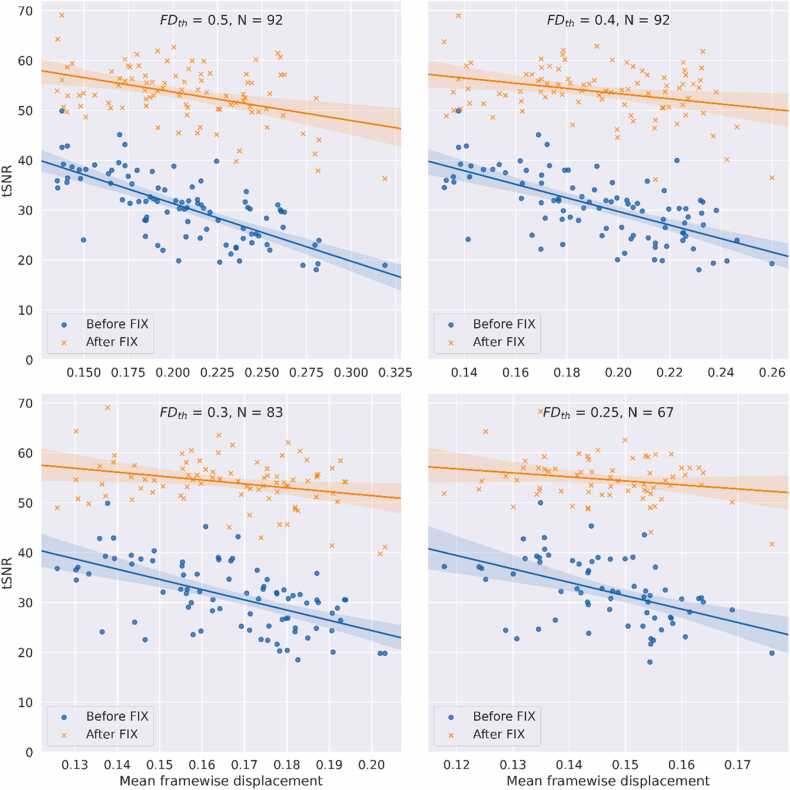
Correlation of temporal SNR with the mean framewise displacement, before and after FIX cleanup, for each of the four framewise displacement censoring thresholds (FD_th_). Mean FD and tSNR were calculated on the first four minutes of data remaining after censoring. FIX cleanup improved tSNR and reduced the correlation of tSNR with mean FD. Stricter censoring thresholds showed the lowest correlation with mean FD and highest average tSNR.

Temporal signal to noise ratio (tSNR) was highly correlated to mean FD before FIX cleanup for all censoring thresholds tested, as shown in Fig. 5. FIX cleanup improved tSNR by 69–75 % across FD thresholds, showing the most dramatic improvements for subjects with higher mean FD, on average. However, even the tSNR for subjects with low mean FD benefitted from the FIX procedure. The correlation between mean FD and tSNR was weaker for stricter thresholds, and weaker yet after the application of FIX, with the correlation reaching insignificance (p > 0.05) for the strictest threshold after FIX. The results are summarized in Table 1.

**Table 1:** Pearson’s correlation coefficient (r) and p-value for tSNR vs mean FD. Correlation strength and significance were reduced with stricter thresholds for volume censoring and after FIX denoising.

Censoring threshold (mm)	Before FIX		After FIX	
	r	p	r	p
0.5	-0.71	2.6 × 10^-16^	-0.42	3.7 × 10^-5^
0.4	-0.64	5.5 × 10^-12^	-0.31	0.0031
0.3	-0.57	1.5 × 10^-8^	-0.29	0.0074
0.25	-0.47	5.4 × 10^-5^	-0.22	0.076

### 3.3 Functional connectivity correlations with FD

**Fig. 6:**
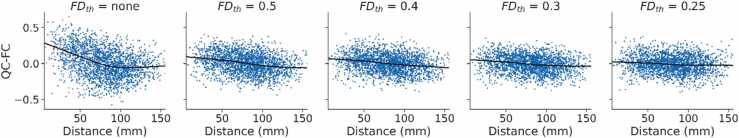
Correlation of functional connectivity node strength with mean framewise displacement for each of the four framewise displacement censoring thresholds (FD_th_) after FIX cleanup, and no set threshold (left, FD_th_ = none), as a function of internodal distance. Lowess fits are shown in black. With no censoring, connectivity strength is greatly overestimated for nodes less than 100 mm apart.

**Fig. 7:**
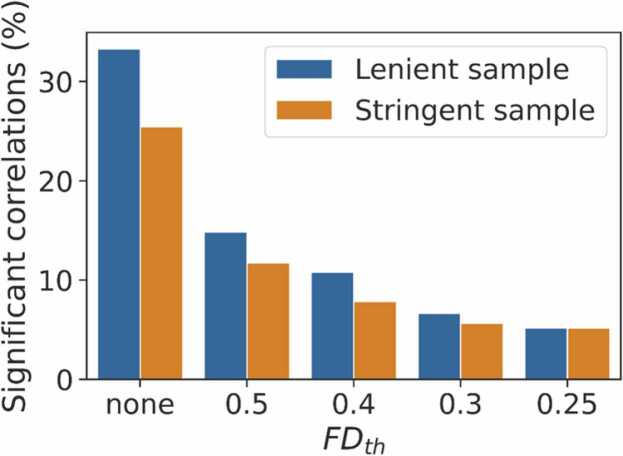
Percentage of functional connectivity nodes whose strength is significantly correlated with mean framewise displacement. Blue bars show the results for all subjects whose data survive each censoring threshold (lenient sample), and orange bars show the results for only the subjects whose data survive the strictest censoring threshold FD_th_ = 0.25 (stringent sample).

Functional connectivity-FD correlations as a function of intranodal distance are shown in Fig. 6. We see an increase in estimated connectivity at short distances, consistent with the results of (Satterthwaite et al., 2012). The dependence of QC-FD correlations on intranodal distance is greatly reduced for all censoring thresholds compared to the no censoring, FIX-only approach. For the two lowest thresholds, this dependence appears eliminated entirely. Fig. 7 shows the percentage of edges significantly correlated with mean framewise displacement (*p* < 0.05). This percentage decreases for each threshold used, with little difference between the two lowest thresholds, 0.3 and 0.25. Because imposing stricter censoring thresholds also excludes more subjects by lowering the quantity of usable data to below our cutoff of 4 min of data, we also plot the same information for only those subjects who survive the strictest threshold (Fig. 7, blue). This analysis allows us to compare the impact of different FD censoring thresholds on functional connectivity correlations in the same set of subjects. Results indicate that there is virtually no difference in number of significant correlations between FD_th_ = 0.3 and FD_th_ = 0.25 with a minimal increase for FD_th_ = 0.4.

### 3.4 BOLD data variance vs framewise displacement

**Fig. 8:**
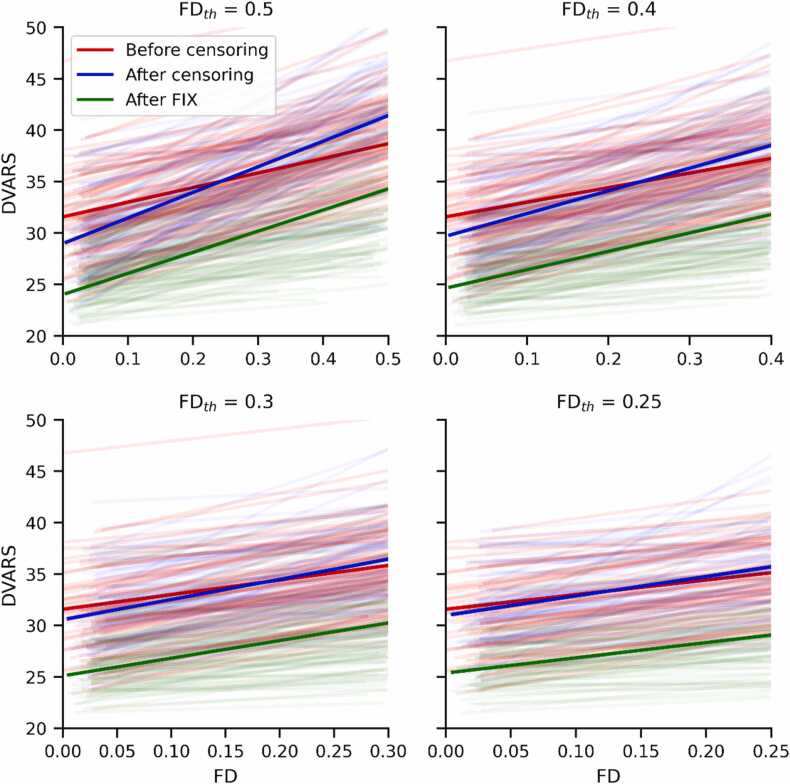
**:** Linear regression of BOLD data variance with framewise displacement for every subject, before censoring, after censoring, and after FIX. Bold lines indicate the linear regressions of DVARS vs FD for all subjects.

An additional means of judging the effectiveness of artifact removal strategies is to plot the BOLD data variance (DVARS) against the framewise displacement for each time point in the data. DVARS is defined as the root mean square of the intensity difference between volumes. Data variance and framewise displacement are expected to be highly correlated, and effective denoising strategies may reduce the strength of this correlation (Moia et al., 2021). Fig. 8 plots this relationship. Faint lines are drawn for the linear regression between DVARS and FD for each subject, and bold lines show the linear regression for the two variables across all subjects. At low levels of censoring, the slope of the linear fit increased from the pre-censored values, presumably because some remaining volumes had moderate framewise displacement but relatively high variance. Before censoring, the r-squared of the regression was 0.66; after censoring the r-squared was reduced to 0.10, 0.06, 0.026, and 0.019 for the thresholds 0.5, 0.4, 0.3, and 0.25 mm respectively. FIX denoising greatly reduced the BOLD data variance for all subjects and censorship thresholds, but slightly increased the r-squared values for the correlation with framewise displacement: 0.16, 0.094, 0.052, and 0.033 for the thresholds 0.5, 0.4, 0.3, and 0.25 mm.

### 3.5 Qualitative analysis of ICA-derived networks

**Fig. 9:**
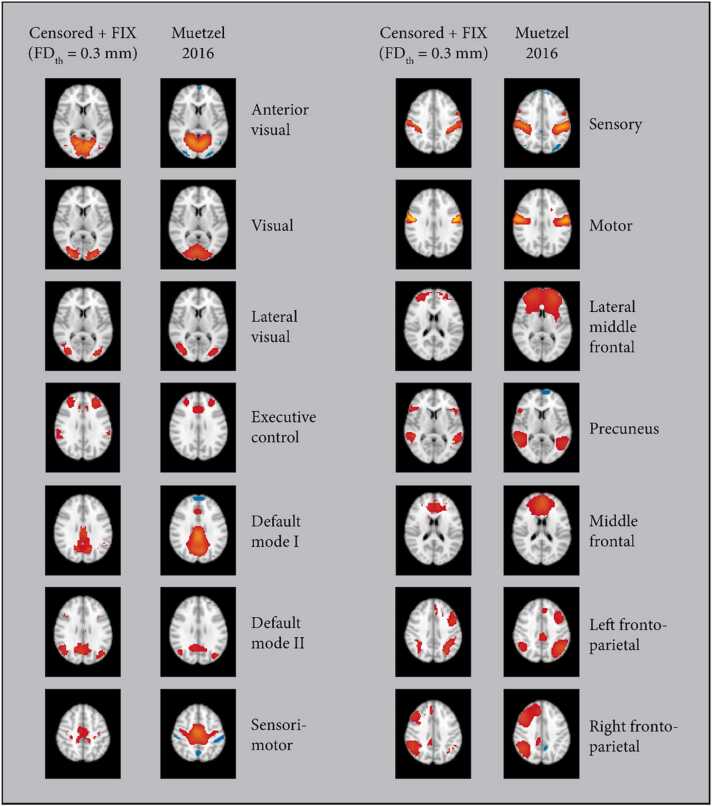
**:** Group ICA results after censoring with a threshold of 0.3 mm mean FD and FIX denoising. Results from a resting state study of 536 6–10 year old children (Muetzel et al., 2016) are shown for comparison. Labels are from Muetzel et al., 2016.

Finally, the performance of the pipeline was qualitatively assessed by visualizing the results of the group ICA. We identified 16 cerebral brain networks from the group MELODIC results, shown in Fig. 9 for FD_th_ = 0.3 mm. These networks were consistent across censoring thresholds, with the executive control and default mode networks each appearing as either a single component or two components, depending on the threshold used. These networks were well-matched to known brain networks from both adults and children (Muetzel et al., 2016, Smith et al., 2009).

## 4 Discussion

Identifying strategies to overcome movement artifact, especially in children, is critical as developmental neuroimaging seeks to elucidate brain mechanisms for typical and atypical developmental processes across a wide range of development. Using a high-movement dataset typical of a healthy cohort in first grade, we examined motion and techniques to mitigate this source of noise. Real-time monitoring enabled us to collect a sufficient number of “clean” frames for each subject. Volume censoring removed the worst motion-corrupted frames, and ICA denoising removed much of the remaining motion artifact. Analysis of data processed with the current pipeline, including tSNR and functional connectivity correlations with FD, suggest that the preprocessed data met rigorous standards for data quality specific to rs-fMRI data while retaining a large majority of subjects (83 % at FD_th_ = 0.3).

Overall, QC-FD correlations compared favorably with other methods reported in the literature (Ciric et al., 2017, Parkes et al., 2018). Parkes et al. compared several pipelines in four different datasets of both normal adult and clinical patient populations. They found the lowest degree of FD-QC correlations using censoring combined with nuisance regression, with between 6.5 % and 11 % of edges significantly correlated with motion. ICA-AROMA, a variation of ICA based cleanup that does not use a training set, fared more poorly, especially when high-motion individuals were not excluded, in which case over 19 % of nodes were significantly correlated with motion. With the current pipeline, less than 10 % of edges correlated with FD at the strictest censoring threshold, FD_th_ = 0.25 (which retained 66 % of the sample), as well as at FD_th_ = 0.3 (which retained 83 % of the sample). These results indicate that a high percentage of the sample can be included in further analysis while maintaining data quality comparable or better than other advanced motion correction strategies.

The current denoising strategies also seem to have a positive impact on data quality beyond the benefit of higher inclusion rates. This is in contrasts with Parkes et al. (2018), who concluded that the primary benefit of scrubbing results from the exclusion of high motion individuals. In order to test this in our data, we looked at only the subjects that survived the strictest subject exclusion threshold, a mean FD threshold of FD_th_ = 0.25 mm. For this group of subjects, there was little difference in the two strictest thresholds, but a much higher number of significant FD-QC correlations for weakest threshold used (FD_th_ = 0.5 mm) and for FIX alone with no censoring. Thus, our data suggest that exclusion of high motion subjects only partially contributes to the data improvement seen with censoring. It should be noted that only 76 % of our subjects would pass the “lenient” threshold used by Parkes et al. (no more than 0.55 mm mean FD), and only 4 % would pass the stringent criteria (specifically, that no more than 20 % of FDs were above 0.2 mm).

While the current efforts to mitigate movement artifact were effective, and compare favorably to other commonly used strategies, we did not conduct an exhaustive analysis of potential strategies. For instance, we did not investigate the effects of using different metrics for censoring, such as BOLD data variance (DVARS), defined as the root mean square intensity difference between volumes. Using DVARS in place of or in addition to FD would remove volumes that show motion artifact in spite of a low framewise displacement (Power et al., 2014). Presumably such artifacts are removed by the FIX denoising process, but further study is needed to test whether DVARS is more effective than FD as a censoring metric when combined with FIX denoising. Imposing a minimum duration for retained segments had no effect on the QC-FD correlations or the group ICA results, except to exclude more subjects whose data now did not meet the required minimum length of time.

We did not correct the motion estimates for respiratory effects. Framewise-displacement motion estimates can be contaminated by physiologically generated artifacts (Fair et al., 2020). These effects can inflate motion estimates, leading to the unnecessary removal of frames due to apparent motion. Applying a notch filter to the motion estimates before censoring may result in a better estimate of true motion and allow more data and subjects to be retained. If the respiration for each subject is monitored, the filter can be customized for each subject. In this study, respiration was not monitored; nevertheless, filtering the framewise displacement measurements based on the average respiratory rates for the population under study may have been beneficial.

Further adjustments to this processing pipeline may improve the results. For example, our FIX training set was considerably noisier than our censored data. Once a value of FD_th_ has been decided for the pipeline, it could be beneficial to train a new dataset using the same censoring threshold, so that the pipeline for the training set matches the pipeline for the functional data to be cleaned. Additionally, we used an adult standard brain for group-level registration. We do not believe this changes our findings regarding pipeline comparisons, but an age-matched standard could be more appropriate for group studies (Muetzel et al., 2016).

In summary, we have shown that it is possible to obtain usable resting-state data despite extreme motion in a group of young, untrained subjects. This involves three key pieces: real-time monitoring of motion, censoring of suspect time points, and ICA-based denoising of concatenated data. Our results show that stricter censoring thresholds reduce the degree of correlation between quality control metrics and measurements of head motion, at the cost of excluding subjects from analysis. Furthermore, we see that this exclusion is not only an issue of losing statistical power, but also may affect the balance of populations within a study because different populations are more likely to exhibit excessive motion. Our results suggest that a framewise displacement threshold of 0.5 mm for volume censoring is likely to be inadequate, as quality control metrics are relatively strongly correlated with head motion. At the opposite extreme, a framewise displacement threshold of 0.25 mm is enough to completely remove the correlation of one quality control measure (tSNR) with mean framewise displacement, at the cost of eliminating 34 % of our subjects. We believe that for this dataset, a framewise displacement threshold of 0.3 mm is sufficient to remove most of the effects of motion without sacrificing too many subjects.

These results have implications for both the collection of new data from challenging population and for the analysis of existing datasets that are highly contaminated by motion. In our study, we needed to collect two minutes of “bad” data on average for every minute of “good” data for each subject. This was driven by a few high-motion outliers; for 70 % of subjects the ratio of “good” to “bad” frames was 1:1 or higher. Use of FIRMM to monitor motion in real time is invaluable with challenging subjects, but if it is unavailable then collecting between two and three times as much data as one expects to need would be a reasonable strategy. With either new or existing datasets, an aggressive use of censoring followed by ICA-based denoising may indeed “save” data that would otherwise be uninterpretable. As discussed, this does come at a cost: not only in the loss of subjects’ data that does not meet the censoring threshold, but also of temporal structure in the data itself. However, it is still possible to investigate brain networks and connectivity as long as such measures are time-invariant, such as Pearson correlations (Ciric et al., 2017, Yan et al., 2013).

## Declaration of Competing Interest

Damien A. Fair is a patent holder on the Framewise Integrated Real-Time Motion Monitoring (FIRMM) software. He is also a co-founder of Nous Imaging Inc. The nature of this financial interest have been reviewed by two committees at the University of Minnesota. They have put in place a plan to help ensure that his research is not affected by the financial interest.

## Data Availability

Data will be made available on request.
